# Socioeconomic Disadvantage Moderates the Association between Peripheral Biomarkers and Childhood Psychopathology

**DOI:** 10.1371/journal.pone.0160455

**Published:** 2016-08-04

**Authors:** Rodrigo B. Mansur, Graccielle R. Cunha, Elson Asevedo, André Zugman, Maiara Zeni-Graiff, Adiel C. Rios, Sumit Sethi, Pawan K. Maurya, Mateus L. Levandowski, Ary Gadelha, Pedro M. Pan, Laura Stertz, Síntia I. Belangero, Márcia Kauer-Sant' Anna, Antônio L. Teixeira, Jair J. Mari, Luis A. Rohde, Euripedes C. Miguel, Roger S. McIntyre, Rodrigo Grassi-Oliveira, Rodrigo A. Bressan, Elisa Brietzke

**Affiliations:** 1 National Institute of Developmental Psychiatry for Children and Adolescents, CNPq, São Paulo, Brazil; 2 Interdisciplinary Lab for Clinical Neurosciences (LiNC), Universidade Federal de Sao Paulo (UNIFESP), Sao Paulo, Brazil; 3 Mood Disorders Psychopharmacology Unit (MDPU), University Health Network, University of Toronto, Toronto, Canada; 4 Institute of Biomedical Research, Pontifícia Universidade Católica do Rio Grande do Sul, Porto Alegre, Brazil; 5 Department of Psychiatry, Universidade Federal do Rio Grande do Sul, Porto Alegre, Brazil; 6 Center for Translational Psychiatry, Department of Psychiatry and Behavioral Sciences, The University of Texas Health Science Center at Houston, Houston, United States of America; 7 Interdisciplinary Laboratory of Medical Investigation, Universidade Federal de Minas Gerais, Belo Horizonte, Brazil; 8 Department of Psychiatry, Universidade de Sao Paulo, Sao Paulo, Brazil; Universite de Bretagne Occidentale, FRANCE

## Abstract

**Background:**

Socioeconomic disadvantage (SED) has been consistently associated with early life mental health problems. SED has been shown to impact multiple biological systems, including the regulation of neurotrophic proteins, immune-inflammatory and oxidative stress markers, which, conversely, have been reported to be relevant to physiological and pathological neurodevelopment This study investigated the relationship between SED, different domains of psychopathology, serum levels of interleukin-6 (IL6), thiobarbituric acid-reactive substance (TBARS) and brain-derived neurotrophic factor (BDNF). We hypothesized that a composite of socioeconomic risk would be associated with psychopathology and altered levels of peripheral biomarkers. In addition, we hypothesized that SED would moderate the associations between mental health problems, IL6, TBARS and BDNF.

**Methods and Findings:**

Using a cross-sectional design, we measured the serum levels of IL6, TBARS and BDNF in 495 children aged 6 to 12. We also investigated socio-demographic characteristics and mental health problems using the Child Behaviour Checklist (CBCL) DSM-oriented scales. SED was evaluated using a cumulative risk model. Generalized linear models were used to assess associations between SED, biomarkers levels and psychopathology. SED was significantly associated with serum levels of IL6 (RR = 1.026, 95% CI 1.004; 1.049, p = 0.020) and TBARS (RR = 1.077, 95% CI 1.028; 1.127, p = 0.002). The association between SED and BDNF was not statistically significant (RR = 1.031, 95% CI 0.997; 1.066, p = 0.077). SED was also significantly associated with all CBCL DSM-oriented scales (all p < 0.05), whereas serum biomarkers (i.e. IL6, TBARS, BDNF) were associated with specific subscales. Moreover, the associations between serum biomarkers and domains of psychopathology were moderated by SED, with stronger correlations between mental health problems, IL6, TBARS, and BDNF being observed in children with high SED.

**Conclusions:**

In children, SED is highly associated with mental health problems. Our findings suggest that this association may be moderated via effects on multiple interacting neurobiological systems.

## Introduction

In children and adolescents, mental health problems are highly prevalent, debilitating and one of the main predictors of adult mental disorders [1–5]. It is well established that childhood psychopathology emerges in the context of an intricate relation between genetic and environmental risk factors [6–8]. Among these environmental risk factors, socioeconomic disadvantage (SED) has been described as one of the major contributors for the development and persistence of mental health problems [9–13]. Epidemiological and clinical evidence indicates that SED is associated with multiple dimensions of psychopathology, with more robust effects on externalizing problems, such as aggressive and delinquent behaviors, and a less robust, but still significant, association with internalizing symptoms, such as anxiety and depression [10–12, 14].

Several mechanisms have been proposed to explain the effects of SED on psychopathology. Low socioeconomic position is often associated with material deprivation, as well as with residence in neighborhoods where crime and substance abuse tend to be more prevalent; and educational/economic opportunities less available [15–17]. Exposure to chronic stress, frequently, but not exclusively, related to the experience of “social defeat”, or the experience of being excluded or isolated, has also been conceptualized as key factor [18, 19]. More recently, an association between SED and the neural substrates of psychopathology has been highlighted. Neuroimaging studies have reported and association between SED and alterations in brain structure and function, characterized, for example, by a decreased volume in the prefrontal cortex and its subdivisions (e.g. orbitofrontal cortex, anterior cingulate cortex), areas prominently involved in cognitive and emotional processing [13, 20–22]. Longitudinal studies have documented that SED was associated with divergent neurodevelopmental trajectories, with children from low socioeconomic background having slower gray matter growth during childhood [23, 24].

From a molecular perspective, neurotrophic proteins, immune-inflammatory and oxidative stress markers have been consistently reported to be associated with brain structure and function and to be relevant to physiological and pathological neurodevelopment [25, 26]. Associations between alterations in these systems have been reliably described in children and adults, across disparate mental disorders [27–32]. Moreover, convergent evidence indicates that early life SED is independently and strongly associated with inflammation in children [33], as well as prospectively in adults [34–37]. Conversely, serum levels of brain-derived neurotrophic factor (BDNF) and functional variations of the BDNF gene were also shown to be affected by SED [38, 39].

We recently demonstrated that SED is associated with general psychopathology, independently from co-occurring risk factors (i.e. parental mental disorders, perinatal complications) (Mansur et al., unpublished data). Moreover, we also documented that exposure to environmental risk factors moderates the association between IL6 and general psychopathology [40]. However, the association between peripheral biomarkers and mental health problems is not completely understood, especially regarding the factors that mediate and/or moderate this relationship. Considering that SED could potentially impact the neural systems that underlie psychopathology, as well modulate systemic adaptations (e.g. immune and endocrine changes), it is possible that this factor could, at least partially, explain the association between SED and different domains of psychopathology. Herein we sought to extend results from these previous studies by evaluating the impact of SED on serum IL6, BDNF and the marker of lipid peroxidation thiobarbituric acid-reactive substance (TBARS). We also aimed to assess the impact of SED on the association between biomarkers and dimension of psychopathology. We hypothesized that (1) SED would be associated with altered levels of IL6, TBARS and BDNF levels; and (2) SED would moderate the association between biomarkers and psychopathology, wherein the correlation between biomarkers and dimensions of psychopathology would be stronger in children with exposure to high SED.

## Methods

### Participants

The sample herein is part of the High Risk Cohort Study for Psychiatric Disorders Study, which has been reported elsewhere [41]. From the total cohort of 2,512 subjects, 1,004 children were invited to participate in enriched imaging/biomarker cohort. A total of 741 subjects completed the imaging procedures and 495 children provided valid blood samples for the study herein. Primary reasons for missing blood samples were: caregiver refusal, children refusal and technical complications during blood processing procedures. Written informed consent was provided by all parents of participants, and verbal consent was obtained from all children. The study was approved by the Ethics Committee of the Universidade de São Paulo (IORG0004884). All families were invited for an appointment with a trained psychologist and social worker in case they were interested in receiving the results of the study evaluation. All children identified as being under the need of care were referred for clinical evaluation. Situations involving serious risk of physical or psychological harm received special attention in accordance to competent authorities’ guidelines.

### Measurements

#### Environmental risk factors

Questions about risk factors were determined after a critical review of the extant literature that has primarily reported on risk factors for mental disorders [41] and included inquiries about demographic and social factors (e.g. socio-economic status, parental education). We created a cumulative risk index, conceptualized as each individual’s cumulative exposure to a set of indicators of SED, according to previous studies [42–46]. Definitions and descriptive statistics of risk factors indicators are reported in Table 1. Each indicator was weighted equally and summed. For analyses of interaction we created a dichotomous variable for high exposure, defined as exposure to 2 or more indicators of SED.

**Table 1:** Definitions and descriptive statistics.

Socioeconomic Disadvantage Index		
Label	Definition	n, %
1. Social Welfare	Receiving governmental social assistance (e.g. *Bolsa Família* [Family Allowance])	113 (22.8%)
2. Low Income	Income from both parents in the lower 25^th^percentile of the sample	115 (23.2%)
3. Single Parent	Living in a single-parent household	110 (22.2%)
4. Unemployment	Having at least 1 parent who is currently unemployed	48 (9.7%)
5. Low Educational Achievement	Both parents with incomplete primary education	45 (9.1%)

#### Child Behavior Checklist (CBCL)

Psychopathology was assessed dimensionally; using the CBCL, which is a parent-report questionnaire that assesses various behavioral and emotional problems. The CBCL is a widely used standardized measure of maladaptive behavior and emotional complications in individuals between ages 4 and 18 [47, 48]. For the study herein, we used the DSM-oriented scales (i.e. depressive problems, anxiety problems, somatic problems, attention deficit/hyperactivity problems, oppositional defiant problems and conduct problems), which have good validity and clinical usefulness [49, 50].

#### Blood samples collection and biomarkers assessment

Whole blood samples were obtained from all children. All samples were obtained between 10:00am and 4:00pm. After collection, blood was allowed to clot by leaving it undisturbed at room temperature and then serum extracted after blood had been processed at 1,000–2,000 x g for 10 minutes in a refrigerated centrifuge. Serum was kept at −80°C until further analyzed. As the samples were labeled with numbers, without any group identification, the investigators were blinded for all procedures.

BDNF serum levels were measured with sandwich-ELISA, using a commercial kit according to the manufacturer's instructions (Milipore, USA). For assessment of oxidative stress, serum levels of malondialdehyde (MDA), a product of lipid peroxidation, were measured by the TBARS (thiobarbituric acid reactive substances) method [51]. Serum IL6 levels were measured by flow cytometry using the Cytometric Bead Array (CBA) Flex Set Kit (BD Biosciences, San Jose, CA) (Cat. #558276). Acquisition was performed with a FACSCanto II flow cytometer (BD Biosciences, San Jose, CA). The instrument has been checked for sensitivity and overall performance with Cytometer Setup and Tracking beads (BD Biosciences) prior to data acquisition. Quantitative results were generated using FCAP Array v1.0.1 software (Soft Flow Inc., Pecs, Hungary).

### Statistical analyses

All statistical analyses were conducted using SPSS software for Windows (version 23.0). For the comparison of demographic and clinical data, the independent samples t-test was used for quantitative variables; the Chi-square test was used for categorical variables. Generalized linear models were used to assess associations between SED, biomarkers levels and psychopathology. We used linear, Poisson (for count data, e.g. CBCL scales) and gamma (for positively skewed distribution, e.g. serum TBARS and IL6 levels) distributions, as appropriate. Interactions between SED and biomarkers were assessed by adding the product term (i.e. SED*IL6) to the tested models. Due to the non-linearity of the models, the estimated β coefficients were transformed into rate ratio (RR) estimates. *Post hoc* correction to control for the false discovery rate was applied according to the Benjamini Hochberg procedure [52].

## Results

### Sample characteristics

The mean age was 10.06 years (SD 1.88) (males 9.94 years, SD 1.91; females 10.19 years, SD 1.85). 45.1% of the study population were female and 58% of the study population were Caucasian. SED index range was 0–5 and the median was 2.0 (SD 1.05); 263 (53.1%) children were classified as high exposure (i.e. exposed to 2 or more social indicators). Twenty-eight children (5.7%) were not exposed to any SED factor, 204 children (41.2%) were exposed to 1 SED factor, 152 children (30.7%) were exposed to 2, 77 (15.6%) were exposed to 3, 27 (5.5%) were exposed to 4; and 7 (1.4%) were exposed to 5 SED factors. No significant correlation was found between the SED index and, respectively, age (r = 0.043, p = 0.339), gender (p = 0.826) or ethnicity (p = 0.644). There was also no association between the binary indicator of high SED and age (p = 0.532), gender (p = 0.925) and ethnicity (p = 0.650).

Means, SDs and ranges for the CBCL DSM-oriented scales were, respectively: depressive problems 4.43, 4.16, 0–22; anxiety problems 3.84, 2.63, 0–11; somatic problems 2.11, 2.08, 0–11; attention deficit/hyperactivity problems 5.86, 3.82, 0–14; oppositional defiant problems 3.98, 2.81, 0–10; and conduct problems 3.42. 4.20, 0–20. As for specific diagnosis, 76 children (15.4%) were diagnosed with an anxiety disorder, 25 (5.1%) with a mood disorder, 61 (12.3%) with attention deficit/hyperactivity disorder, 38 (7.7%) with oppositional/conduct disorders, 3 (0.6%) with tic disorders and 4 (0.8%) with eating disorders).

### Socioeconomic disadvantage and peripheral biomarkers

Serum IL6 median was 2.58 pg/ml (interquartile range [IQR] 2.22–2.91), TBARS median was 14.04 pg/ml (IQR 9.63–21.72) and BDNF median was 26.04 pg/ml (IQR 20.12–33.54). There was no correlation between age, IL6 (r = 0.059, p = 0.190), TBARS (r = -0.016, p = 0.723) and BDNF levels (r = 0.065, p = 0.151). There was also no effect of ethnicity (p = 0.058, p = 0.708, p = 0.537, respectively); BDNF levels were higher in female participants (p = 0.002), but no effect of gender were observed on serum IL6 (p = 0.060) or TBARS (p = 0.695).

There was a positive correlation between the SED index, serum IL6 (r = 0.121, p = 0.007), TBARS (r = 0.145; p = 0.001) and BDNF levels (r = 0.098, p = 0.022). After adjustments for age, gender and ethnicity, the association between SED, IL6 (RR = 1.026, 95% CI 1.004; 1.049, p = 0.020) and TBARS remained significant (RR = 1.077, 95% CI 1.028; 1.127, p = 0.002), whereas there was a trend for BDNF (RR = 1.031, 95% CI 0.997; 1.066, p = 0.077).

### Socioeconomic disadvantage, peripheral biomarkers and psychopathology

Table 2 shows that SED was positively associated with all CBCL scales when analyzed separately (Model 1) and together with the biomarkers (Model 2). The biomarkers had somewhat distinct patterns of associations with the different scales, with IL6 being more strongly associated with scores on the depressive and anxiety scales; and TBARS being associated with the anxiety and conduct scales. BDNF was only associated with the anxiety, attention and conduct scales when analyzed separately.

**Table 2:** Associations between socioeconomic disadvantage, IL6, TBARS and BDNF; considered separately (Model 1) and together (Model 2). All analyses included age, gender and ethnicity as covariates.

	Model 1			Model 2		
	RR	95% CI	Adj. p-value*	RR	95% CI	Adj. p-value*
Depressive Problems						
SED	**1.118**	**1.080; 1.158**	**< 0.001**	**1.120**	**1.077; 1.164**	**0.004**
IL6	**1.133**	**1.075; 1.194**	**0.005**	**1.142**	**1.080; 1.208**	**< 0.001**
TBARS	1.003	0.999; 1.008	0.242	1.000	0.996; 1.005	0.965
BDNF	1.002	0.998; 1.006	0.392	0.999	0.995; 1.004	0.761
Anxiety Problems						
SED	**1.007**	**1.033; 1.123**	**0.002**	**1.060**	**1.016; 1.106**	**0.018**
IL6	**1.145**	**1.081; 1.213**	**< 0.001**	**1.125**	**1.060; 1.194**	**< 0.001**
TBARS	**1.007**	**1.002; 1.012**	**0.012**	1.005	1.001; 1.010	0.074
BDNF	**1.005**	**1.001; 1.011**	**0.044**	1.003	0.998; 1.008	0.282
Somatic Problems						
SED	**1.081**	**1.022; 1.143**	**0.016**	1.063	1.005; 1.126	0.057
IL6	**1.125**	**1.038; 1.219**	**0.012**	**1.103**	**1.015; 1.198**	**0.037**
TBARS	**1.008**	**1.002; 1.015**	**0.022**	1.006	0.999; 1.013	0.089
BDNF	1.004	0.998; 1.010	0.282	1.002	0.995; 1.008	0.681
Attention Deficit/Hyperactivity Problems						
SED	**1.099**	**1.062; 1.136**	**0.004**	**1.089**	**1.052; 1.126**	**0.003**
IL6	**1.067**	**1.017; 1.120**	**0.019**	1.043	0.991; 1.097	0.152
TBARS	**1.005**	**1.001; 1.009**	**0.021**	1.003	0.999; 1.007	0.146
BDNF	**1.005**	**1.001; 1.008**	**0.032**	1.003	0.999; 1.007	0.179
Oppositional Defiant Problems						
SED	**1.094**	**1.050; 1.140**	**< 0.001**	**1.085**	**1.041; 1.131**	**< 0.001**
IL6	1.061	1.000; 1.126	0.080	1.043	0.981; 1.109	0.236
TBARS	**1.006**	**1.002; 1.011**	**0.019**	1.005	1.000; 1.009	0.089
BDNF	1.002	0.998; 1.007	0.392	1.001	0.996; 1.005	0.844
Conduct Problems						
SED	**1.289**	**1.237; 1.343**	**< 0.001**	**1.276**	**1.223; 1.330**	**< 0.001**
IL6	1.061	0.995; 1.132	0.108	1.004	0.937; 1.077	0.915
TBARS	**1.011**	**1.006; 1.016**	**< 0.001**	**1.007**	**1.002; 1.012**	**0.002**
BDNF	**1.006**	**1.002; 1.011**	**0.019**	1.003	0.999; 1.008	0.224

* Adj. p-value: Benjamini Hochberg *post hoc* corrected p-value.

RR: rate ratio; CI: confidence interval; SED: socioeconomic disadvantage, IL6: interlukin-6; TBARS: thiobarbituric acid-reactive substance; BDNF: brain-derived neurotrophic factor.

**Fig 1:**
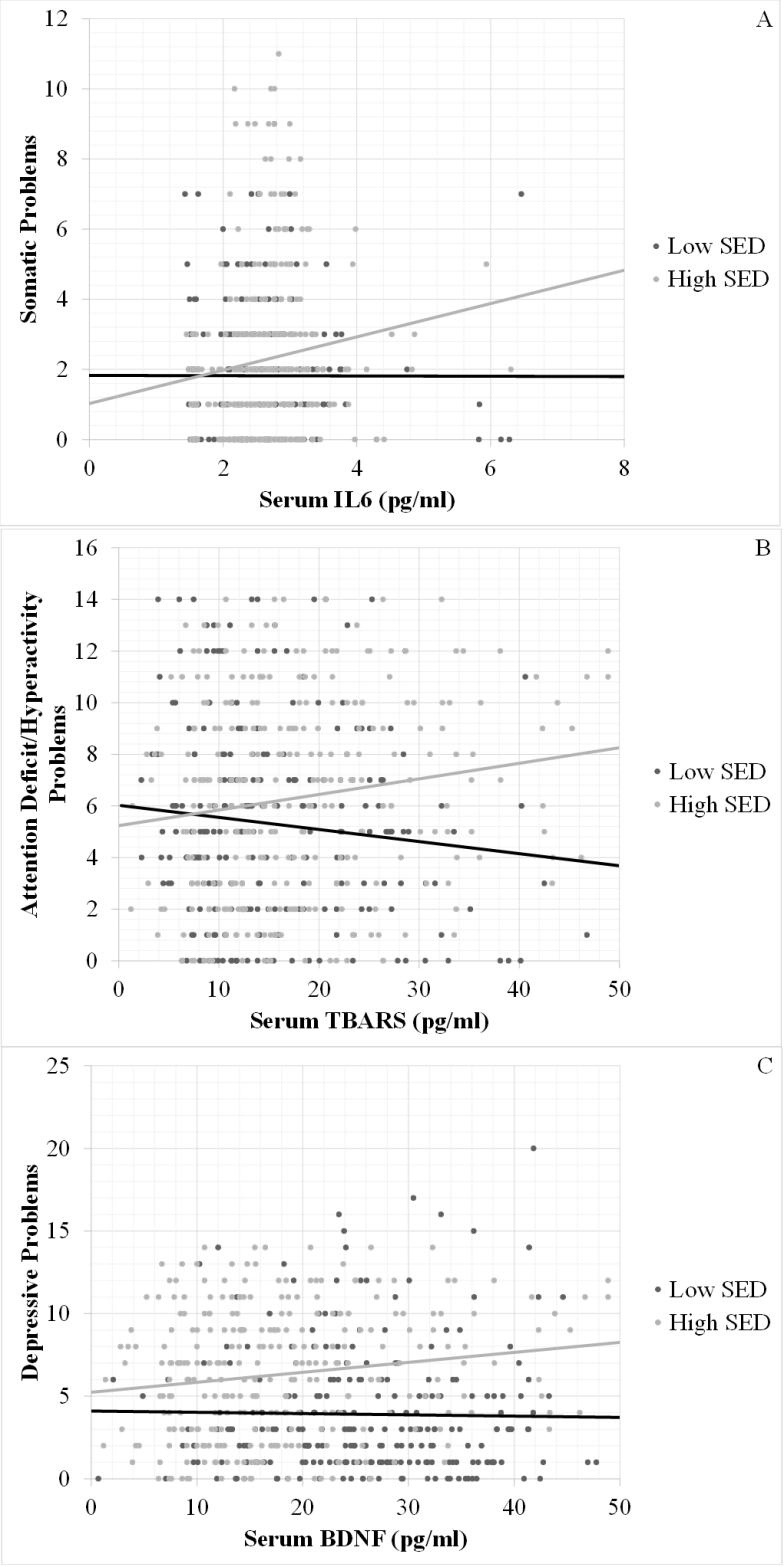
Mental health problems and peripheral biomarkers within socioeconomic disadvantage exposure subgroups. Correlations between (A) somatic problems and serum IL6; (B) attention deficit/hyperactivity problems and serum TBARS and (C) depressive problems and serum BDNF; within socioeconomic disadvantage exposure subgroups. SED: socioeconomic disadvantage; IL6: interlukin-6; TBARS: thiobarbituric acid-reactive substance; BDNF: brain-derived neurotrophic factor.

Interaction analyses indicated a significantly positive interaction between high SED and BDNF on depressive problems, indicating that a positive correlation between BDNF and depressive problems was only positive in the children from the high SED group (Fig 1). The moderating effect of SED on the association between BDNF and psychopathology was specific to depressive problems, as the results were non-significant for measures on other scales (Table 3). Moderating effects on IL6 and TBARS were, on the other hand, pleiotropic, as it was significant in almost all subscales, with the only exceptions being depressive and oppositional defiant problems, for IL6, and somatic problems, for TBARS (Table 3). All of the significant interactive effects with IL6 and TBARS were positive, indicating a stronger correlation between biomarkers and psychopathology in children from the high SED group, compared to children in the low SED group (Fig 1).

**Table 3:** Effects of interactions between high socioeconomic disadvantage and peripheral biomarkers on CBCL scales. All analyses included age, gender and ethnicity as covariates.

	RR	95% CI	Adj. p-value*	RR	95% CI	Adj. p-value*
	Depressive Problems			Attention Deficit/Hyperactivity Problems		
IL6	1.124	1.010; 1.251	0.055	**1.118**	**1.017; 1.230**	**0.038**
TBARS	**1.022**	**1.012; 1.031**	**< 0.001**	**1.020**	**1.012; 1.028**	**< 0.001**
BDNF	**1.013**	**1.005; 1.021**	**0.006**	1.004	0.997; 1.011	0.373
	Anxiety Problems			Oppositional Defiant Problems		
IL6	**1.193**	**1.065; 1.336**	**0.006**	1.092	0.973; 1.225	0.185
TBARS	**1.014**	**1.004; 1.023**	**0.014**	**1.020**	**1.010; 1.029**	**< 0.001**
BDNF	1.005	0.996; 1.014	0.298	1.001	0.993; 1.010	0.771
	Somatic Problems			Conduct Problems		
IL6	**1.207**	**1.031; 1.413**	**0.036**	**1.198**	**1.048; 1.369**	**0.019**
TBARS	1.003	0.991; 1.016	0.681	**1.033**	**1.021; 1.044**	**0.003**
BDNF	1.006	0.995; 1.018	0.355	1.003	0.993; 1.012	0.667

* Adj. p-value: Benjamini Hochberg *post hoc* corrected p-value.

RR: rate ratio; CI: confidence interval; IL6: interlukin-6; TBARS: thiobarbituric acid-reactive substance; BDNF: brain-derived neurotrophic factor.

## Discussion

Our results indicate that SED is robustly associated with multiple domains of psychopathology. The strongest association in our sample was with conduct problems; an association with anxiety symptomatology was also significant [11, 53]. Evidence indicates that familial context (i.e. parents educational level or occupational status) and residence in deprived neighborhoods, with more exposure to deviant peer behavior and lower social support, are more related to externalizing problems [54, 55]. Internalizing symptoms, on contrast, would be more, but not exclusively, associated with individual temperament and genetic vulnerabilities [12, 56].

Serum biomarkers also had differential associations with mental health problems, with the inflammatory cytokine IL6 being more strongly associated with the internalizing dimension (i.e. depressive, anxiety and somatic problems) and the oxidative stress marker TBARS with externalizing symptoms (i.e. attentional, oppositional and conduct problems). The neurotrophin BDNF had, in comparison, weaker associations, especially in the models that analyzed all the variables together. Effect sizes were relatively small, although were largely consistent with previous mechanistic studies [29, 33]. Considering the complexity of mental illnesses etiology, which involves multiple interacting genetic, environmental and biological factors; large effect sizes are unlikely to be detected; therefore the magnitude of the associations described in this community-based study are noteworthy and possibly indicative of clinical relevance.

Evidence on putative specific effects of different pathophysiological pathways is scarce. There are reports of positive correlations between plasma markers of oxidative stress and attention deficit hyperactivity disorder, as well as aggressive behavior in adults [57, 58]. Oxidative stress induces damage to nucleic acids or lipids, which has the potential to impair basic cellular/neuronal functions [59]. Indeed, there is evidence that lipid peroxidation is associated with white matter damage, which could potentially affect the circuits that regulate aggressive/confrontational behavior [60, 61]. Consistent with our results, alterations in inflammatory markers, including an increase in serum IL6, have been observed in children and adolescents with major depressive disorder [32]. The relationship between inflammation and mood has been extensively studied, with potential mechanisms including, but not limited to, effects on monoamine levels through activation of indoleamine 2,3-dioxygenase (IDO), which degrades tryptophan, and pathologic microglial cell activation [62, 63].

Peripheral biomarkers were positively correlated with SED, with higher levels of IL6, TBARS and BDNF being found in children with low socioeconomic position. As hypothesized, SED moderated the association between IL6, TBARS, BDNF and domains of psychopathology. All significant interactions were in the same direction, with a stronger association between peripheral markers and mental health problems in children exposed to high SED. Interestingly, we observed an interaction between SED and BDNF on depressive problems, even though there was no significant association between BDNF and depressive symptoms, indicating that BDNF’s relationship with this domain of psychopathology may be fully dependent of socioeconomic position. Associations between IL6 and internalizing problems, as well as between TBARS and externalizing symptoms, were also modified by the presence of SED. These results indicate that the differential effects of SED on each domain of psychopathology may be subserved by differential activations of neurobiological pathways.

Conceptually, the accumulation of multiple adverse conditions (e.g. SED) may lead to several different types of emotional or behavioral outcomes, which has been termed multifinality [64]. This model has been empirically supported [53, 65, 66]; nonetheless, there is evidence that specific developmental trajectories are more likely than others. For example, some psychopathology constructs are more likely to predict themselves (i.e. homotypic continuity), whereas some domains are more likely to predict others (i.e. heterotypic continuity). Evidence indicates that conduct problems are mostly stable over time; oppositional/defiant problems, instead, are stronger predictors of affective and attentional problems [66, 67]. These developmental pathways are dynamically influenced by genetic and non-genetic factors [6, 68, 69]. Interestingly, this transition from oppositional problems toward mood/anxiety symptoms seems to be partially mediated by environmental risk factors [66, 70]. Our data suggest that SED’s relationship with different neurobiological substrates, likely determined by each individual genetic vulnerabilities and/or previous or co-occurring exposure to other environmental factors, accounts, at least partially, to its differential associations with disparate domains of psychopathology.

Nevertheless, the principle of equifinality, which refers to a diversity of pathways leading to same phenotype, may also apply (62). Low socioeconomic position is frequently correlated with other risk factors, including, but not limited to, parental mental disorders and exposure to perinatal complications [11, 71–73]. We recently reported, using data from this same sample, that SED is associated with parental mental disorders, but that its association with general psychopathology was independent and did not interact with familial mental illness (Mansur et al., unpublished data), a finding that is consistent with other studies [10]. However, we separately documented an interaction between SED and parental psychopathology on IL6 levels[40], indicating that the results described herein may not represent an isolated effect of SED. Our sample size and study design does not allow the disentangling of multiple risk factors’ effects, therefore, these findings need to be replicated and refined, with consideration for reciprocate and interacting associate factors, in larger, prospective samples.

This study has limitations that limit inferences and interpretations of the data. The cross-sectional design precludes conclusions about causality. It is not possible to determine, based on our data, whether exposure to SED or alterations in serum biomarkers precede the onset of psychopathology. We used a cumulative composite of SED, therefore other important determinants of environmental factors impact, such as extent and timing of exposure, were not directly assessed. Our SED index weighted equally all components. The studied factors are not interchangeable and may impact different etiological pathways; it is also possible that different combinations may have divergent effects [74, 75]. Moreover, as there are no longitudinal studies evaluating the markers assessed in this study, there are questions about their stability over time. We collected the samples in a relatively narrow period of the day; however, it is possible that the biomarker’s levels were affected by temporal and contextual factors. Nonetheless, our study also has a number of strengths. Our study population was derived from a large, community-based sample, enriched for the presence of psychopathology. We used a multi-informant clinical evaluation with validated instruments, thus obtaining data directly from parents and limiting rater and information biases. Finally, we simultaneously assessed a range of dimensional domains of psychopathology, which provides more insight on the diverse effects of SED.

In summary, SED was associated with disparate domains of psychopathology in children, as well as with increased serum levels of IL6, TBARS and BDNF. In addition, SED was also shown to moderate the association between IL6, TBARS and BDNF, and mental health problems, suggesting that SED’s different associations with psychopathology are, at least partially, related to its engagement of different neurobiological pathways. Prospective evaluation of this cohort may provide further information about the interaction between SED, serum biomarkers, psychopathology, and the onset of psychiatric disorders.

## Supporting Information

S1 Dataset Dataset. (SAV)
